# Syllidae (Annelida, Polychaeta) from the Caribbean coast of Venezuela

**DOI:** 10.3897/zookeys.117.858

**Published:** 2011-07-08

**Authors:** Ildefonso Liñero-Arana, Oscarn Díaz Díaz

**Affiliations:** Instituto Oceanográfico de Venezuela, Universidad de Oriente, Ofic. 208. Av. Universidad, Cerro Colorado, Cumaná, Venezuela

**Keywords:** Annelida, Polychaeta, Syllidae, Venezuela, Caribbean

## Abstract

Venezuela possesses a great variety of coastal environments allowing for a high diversity of marine species. However, systematic studies on marine invertebrates are scarce, especially on polychaetes. The family Syllidae is poorly known, and only 14 genera and 42 species have been reported from this country. A total of 13 genera and 26 species the Syllidae were identified from benthic samples collected on different substrata of the northeastern coast of Venezuela. Of these, seven genera and 16 species constitute new records for Venezuela: *Odontosyllis guillermoi*, *Syllides floridanus*, *Salvatoria clavata*, *Salvatoria limbata*, *Sphaerosyllis longicauda*, *Parapionosyllis longicirrata*, *Trypanosyllis parvidentata*, *Trypanosyllis vittigera*, *Opisthosyllis* sp., *Syllis amica*, *Syllis armillaris*, *Syllis gracilis*, *Syllis pseudoarmillaris*, *Syllis vittata*, *Parasphaerosyllis indica* and *Myrianida convoluta*.

## Abstract (es)

Venezuela posee una amplia variedad de ambientes costeros que permiten la existencia de una elevada diversidad de especies. Sin embargo, los estudios sobre la sistemática de los invertebrados marinos son escasos, especialmente en poliquetos. La familia Syllidae es muy poco conocida en este país, puesto que sólo han sido registrados 14 géneros y 42 especies. En muestreos realizados en diferentes tipos de substrato, se han recolectado 13 géneros y 26 especies, de los cuales 7 géneros y 16 especies constituyen nuevos registros para Venezuela: *Odontosyllis guillermoi*, *Syllides floridanus*, *Salvatoria clavata*, *Salvatoria limbata*, *Sphaerosyllis longicauda*, *Parapionosyllis longicirrata*, *Trypanosyllis parvidentata*, *Trypanosyllis vittigera*, *Opisthosyllis* sp., *Syllis amica*, *Syllis armillaris*, *Syllis gracilis*, *Syllis pseudoarmillaris*, *Syllis vittata*, *Parasphaerosyllis indica* y *Myrianida convoluta*.

## Introduction

There have been very few studies done on the benthic macrofauna, especially polychaetes, along the Caribbean coast of South America. Syllidae is one of the most abundant within the polychaete families. It is constituted by about 55 valid genera and approximately 667 species (San Martin 2003), of which a total of 31 genera and 167 species have been described from the Great Caribbean Region (Salazar-Vallejo 1996). In soft and hard bottoms of the eastern coasts of Mexico (Gulf of Mexico and Caribbean), 45 species of syllids were identified by Granados-Barba et al. (2003). In nearby Trinidad and Tobago islands, Syllidae was both the most abundant (70%) and diverse (30 species) family collected from hard bottom substrates (Gobin 2010). In Venezuela, very little is known about this family, and only two systematic studies have been carried out, both by San Martin and Bone (1999, 2001) on *Thalassia testudinum* (Bank & Köning, 1805) meadows. In 1999 these authors described two new species and in 2001 they reported 13 genera and 40 species, of which 35 constituted new records for Venezuela. In this study, syllid species collected from different localities and substrata of the northeastern coast of Venezuela are reported.

## Materials and methods

The examined material belongs to samples from the Benthos Laboratory polychaete collection at the Instituto Oceanográfico de Venezuela, collected from 1984 until the present. Samples were collected manually or by using dredges and corers on different substrata: rocky shores, sandy and muddy bottoms, on dock piles of PVC and in dead of the fire coral *Millepora alcicornis* Linnaeus in Mochima Bay (Isla Larga, Punta León, Ensenada de Reyes, Mangle Quemado, Cabruta, and La Virgen) and the Gulf of Cariaco (Turpialito, Guacarapo and La Bruja), inside sponges *Aplysina fistularis* (Pallas, 1766), *Ircinia felix* (Duchassaing & Michelott, 1864), and *Chondrila nucula* Schmidt, 1862 (Porifera: Demospongiae) in Mochima Bay (Isla Larga, Punta León); from *Rhizophora mangle* Linnaeus root mats covered with the bivalve *Crassostrea rhizophorae* (Gmelin) in La Restinga Lagoon, Margarita Island, from sandy bottoms at the mouth of Bocaripo Lagoon, and as epibionts on tubes of *Americonuphis magna* (Andrews, 1891) from Chacopata Beach (see coordinates and dates of collections in Table 1).

Specimens were fixed in 10% seawater formalin during at least 24 hours and then preserved in 70% ethanol. Microscope slides of specimens were made in glycerine. Measurements were made using an ocular micrometer. Voucher specimens are deposited in the Benthos Laboratory at the Instituto Oceanográfico de Venezuela.

**Table 1.** List of sites (codes, names), coordinates and dates where the syllids were collected.

SITE CODE	SITE NAME	COORDINATES	DATE
	Mochima Bay		
BMC101	Cabruta	10°22'05"N, 64°20'14"W	18/11/01
BMC203	Cabruta	10°22'05"N, 64°20'14"W	05/08/03
BMLV101	La Virgen	10°22'35"N, 64°20'42"W	05/08/03
BMPL197	Punta León	10°22'20"N, 64°20'22"W	22/04/97
BMPL197	Punta León	10°22'20"N, 64°20'22"W	19/07/07
BMPL297	Punta León	10°22'20"N, 64°20'22"W	23/11/97
BMPL398	Punta León	10°22'20"N, 64°20'22"W	28/01/98
BMPL498	Punta León	10°22'20"N, 64°20'22"W	15/05/98
BMPL501	Punta León	10°22'20"N, 64°20'22"W	18/11/01
BMMQ103	Mangle Quemado	10°21'55"N, 64°21'05"W	05/08/03
BMMQ205	Mangle Quemado	10°21'55"N, 64°21'05"W	14/02/05
BMIL197	Isla Larga	10°21'21"N, 64°20'58"W	22/04/97
BMIL297	Isla Larga	10°21'21"N, 64°20'58"W	19/07/97
BMIL397	Isla Larga	10°21'21"N, 64°20'58"W	23/11/97
BMIL498	Isla Larga	10°21'21"N, 64°20'58"W	28/01/98
BMIL598	Isla Larga	10°21'21"N, 64°20'58"W	15/05/98
BMIL602	Isla Larga	10°21'21"N, 64°20'58"W	27/03/02
BMIL803	Isla Larga	10°21'21"N, 64°20'58"W	05/08/03
BMER103	Ensenada de Reyes	10°20'19"N, 64°22'07"W	05/05/03
BMER203	Ensenada de Reyes	10°20'19"N, 64°22'07"W	05/08/03
	Cariaco Gulf		
GCPG198	Guacarapo	10°28'49"N, 64°42'01"W	12/05/98
GCET103	Turpialito	10°26'34"N, 64°01'59"W	12/10/03
GCLB104	La Bruja	10°26'43"N, 63°58'25"W	29/05/04
GCLB205	La Bruja	10°26'43"N, 63°58'25"W	26/07/05
GCPT106	Tocuchare	10°26'26"N, 64°00'46"W	29/05/06
	Peninsula of Araya		
PALB104	Bocaripo lagoon	10°39'36"N, 63°49'25"W	29/05/04
PAPC106	Chacopata beach	10°40'40"N, 63°49'19"W	18/06/06
	Margarita Island		
IMLR102	La Restinga lagoon	10°59'30"N, 64°09'21"W	22/05/02
IMLR202	La Restinga lagoon	10°59'30"N, 64°09'21"W	12/10/06

## Results

### Family Sillydae Grube, 1850. Subfamily Anoplosyllinae Aguado & San Martín, 2009. Genus Odontosyllis Claparède, 1863. Type species: Syllis fulgurans Audouin & Milne Edwards, 1834

#### Odontosyllis enopla

Verrill, 1900

http://species-id.net/wiki/Odontosyllis_enopla

Figs 1.1–1.4

Odontosyllis enopla Hartman 1951:41.–Taylor 1971:205.–Uebelacker 1984:81–82, fig. 76a–g.–San Martín and Bone 2001:611.

##### Material examined.

 GCPG198, (8), fine sand, 2 m depth; BMMQ103, (22), fine to coarse sand, 1 m depth; GCLB205, (17), fine sand, 1 m depth.

##### Description.

 Length to 22.3 mm, width to 1.1 mm. Body with up to 76 chaetigers. Prostomium with anterior pair of eyespots and two pairs of large, lentigerous eyes. Median antenna long; lateral antennae shorter than median one. Nuchal organs as crescent-shaped ridges along posterior margin of prostomium. Occipital flap present. Dorsal cirri alternating in length. Compound falcigers bidentate, with fimbriated sheath between blade and shaft-head (Fig. 1.1). Dorsal simple chaeta, only present on posterior chaetigers (Fig. 1.2). Ventral simple chaeta bidentate (Fig. 1.3). Acicula subdistally enlarged, with numerous serrations encircling the tip (fig. 1.4). Pharynx extending to chaetigers 4–7, with six relatively large teeth, two lateral plates and four smaller ones; proventriculus from chaetigers 5–8 to 9–11, with 41–57 rows of muscle cells. Pygidium with a pair of cirriform anal cirri.

**Figure 1.**
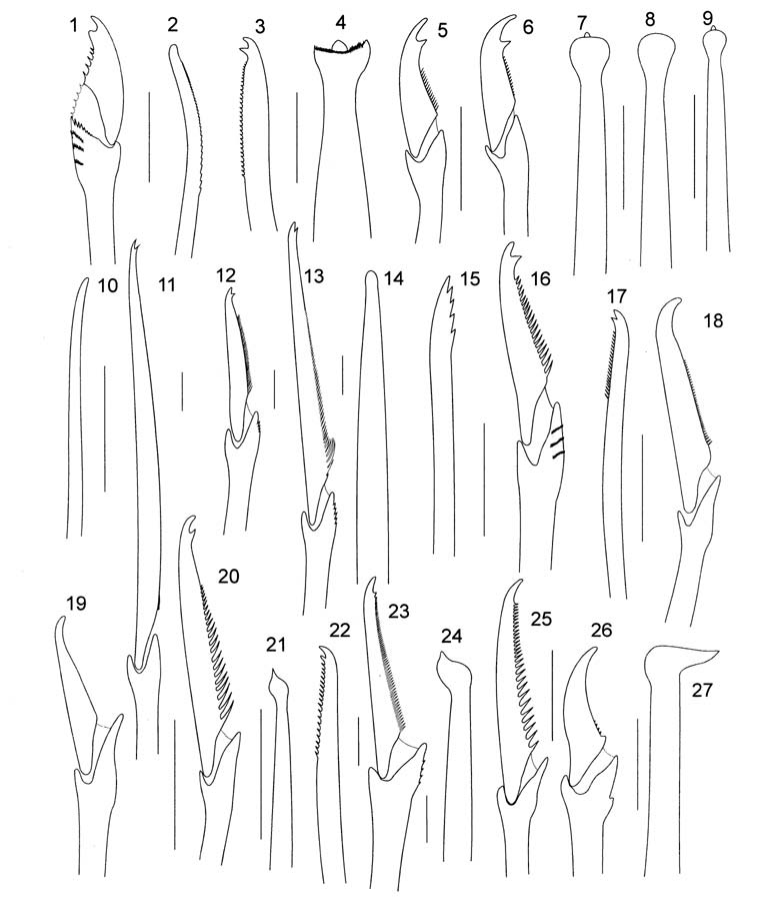
*Odontosyllis enopla* **1** bidentate falciger, midbody chaetiger **2** dorsal simple chaeta, posterior chaetiger **3** ventral simple chaeta **4** acicula. *Odontosyllis guillermoi* **5** bidentate falciger, anterior chaetiger **6** bidentate falciger, midbody chaetiger **7-10** aciculae, anterior chaetiger. *Syllides floridanus* **11** long bidentate falciger, anterior chaetiger **12** short bidentate falciger, midbody chaetiger **13** bidentate falciger from same **14** neuroacicula, midbody chaetiger. *Salvatoria clavata* **15** dorsal simple chaeta, anterior chaetiger **16** ventral bidentate falciger, midbody chaetiger **17** ventral simple chaeta, posterior chaetiger. *Salvatoria limbata* **18** unidentate serrated falciger, midbody chaetiger **19** unidentate smooth falciger from same **20** bidentate falciger, midbody chaetiger **21** acicula from same. *Sphaerosyllis longicauda* **22** dorsal simple chaeta, anterior chaetiger **23** bidentate falciger, midbody chaetiger **24** acicula from same. *Sphaerosyllis piriferopsis* **25** dorsal unidentate falciger, anterior chaetiger **26** ventral unidentate falciger from same **27** acicula, midbody chaetiger (scale bars: 10µm).

##### Distribution.

 Barbados, Bermuda, Gulf of Mexico, Venezuela.

#### Odontosyllis guillermoi

Fukuda & Nogueira, 2006

http://species-id.net/wiki/Odontosyllis_guillermoi

Figs 1.5–1.10

Odontosyllis guillermoi Fukuda and Nogueira 2006:225–229, figs. 1–2.

##### Material examined.

 IMLR102, (18), as epibionts on *Crassostrea rhizophorae*, 0–0.5 m depth.

##### Description.

 Length to 15.3 mm, width to 1.2 mm. Body with up to 76 chaetigers, with two black transverse stripes per segment. Nuchal organs at center of prostomium. Occipital flap rounded with diffuse black spot. Dorsal cirri of chaetiger 1 longer than remainder cirri. Dorsal cirri in median region alternately long and short. Bidentate falcigers serrated, with distal tooth hooked and slightly longer than subdistal one on anterior chaetigers (Fig. 1.5), much longer from midbody (Fig. 1.6). Dorsal simple chaeta, only present on posterior chaetigers, with rounded tip and short subdistal spines. Ventral shorter simple chaeta may be present. Anterior parapodia with four aciculae subdistally inflated (Figs. 1.7-1.9), and slender, pointed acicula (fig. 1.10). Parapodia from middbody region with 2-3 aciculae, on posterior chaetigers a single acicula subdistally inflated. Pharynx extending through 9–10 chaetigers, trepan with 6 ventral teeth and 2 lateral plates. Proventriculus extending through 5–10 chaetigers, with numerous rows of muscle cells. Pygidium with a pair of anal cirri.

##### Distribution.

 Sao Paulo (Brazil), Margarita Island (Venezuela).

#### Genus Syllides Örsted, 1845. Type species: Syllides longocirrata Örsted, 1845

##### Syllides floridanus Perkins, 1981

http://species-id.net/wiki/Syllides_floridanus

Figs 1.11–1.14

Syllides floridanus Perkins 1981:1151–1155, figs. 31–32.–Uebelacker 1984:45–47, fig. 38a–d.–San Martín 1990:609.

###### Material examined.

 BMMQ103, (8), fine sand, 4 m depth; BMC203, medium sand, 2 m depth. GCET103, (10), coarse sand, 1 m depth.

###### Description.

 Length to 3.1 mm, width to 0.4 mm. Body small, slender; complete specimens with up to 22 chaetigers. Prostomium rounded, with three pairs of lentigerous eyes Median and lateral antennae digitiform, median slightly longer, about as long as prostomium plus palps. Palps short, triangular, fused basally. Dorsal tentacular cirri longer than median antenna, ventral tentacular cirri shorter than dorsal ones. Dorsal cirri of chaetigers 1 and 2 slightly wrinkled. Articulated dorsal cirri from chaetiger 3, with 14–19 articles. Dorsal simple chaeta pointed and serrated, from chaetiger 1. Compound falcigers bidentate, with long and short blades serrated (Fig. 1.11, 1.12), basal serrations longer and coarser on some blades (Fig. 1.13). Noto and neuroacicula slender, the alter with blunt end (Fig. 1.14). Pharynx extending through 5–7 chaetigers, with about 10 marginal papillae. Proventriculus extending through 5 chaetigers, with 38–44 rows of muscle cells. Pygidium with a pair of cirriform anal cirri.

###### Distribution.

 East coast of Florida, Gulf of Mexico, Venezuela.

#### Subfamily Exogoninae Langerhans, 1879. Genus: SalvatoriaMcIntosh, 1885. Type species: Salvatoria kerguelensis McIntosh, 1885

##### Salvatoria clavata

(Claparède, 1863)

http://species-id.net/wiki/Salvatoria_clavata

Figs 1.15–1.17

Grubea clavata Fauvel 1923:296–298, fig. l14a–e.
Brania clavata Pettibone 1963:133, fig. 35b**.–**Imajima 1966:393, fig. la–g.–Taylor, 1971:198–200.–Gardiner 1976:130, fig. 10l–n.–Uebelacker 1984:16–19, figs. 10a–e.–Russell 2007:51–52.
Salvatoria clavata San Martín 2003:176–181, figs. 89–93.–Gobin 2010 (list only).

###### Material examined.

 BMIL197, (23); BMIL498, (18); BMPL398, (2), all specimens associated with *Aplysina fistularis*, 1–3 m depth; BMIL397, (50); BMPL398, (13); BMPL501, (14), all specimens associated with *Ircinia felix*, 1–2m depth; GCPG198, (6), on artificial substrate (PVC pipes), 1 m depth.

###### Description.

 Length to 3.8 mm width to 0.4 mm. Body small, slender; complete specimens with up to 33 chaetigers. Prostomium with two pairs of lentigerous eyes on posterior region of prostomium and two ocular spots near posterior base of palps. Subulate enlarged antennae. Palps fused dorsally. Tentacular and dorsal cirri fusiform. Dorsal tentacular cirri about twice length of ventral ones. Ventral cirri digitiform. Dorsal simple chaeta bidentate with subdistal serrations (Fig. 1.15). Compound bidentate falcigers with serrated blades (Fig. 1.16). Bidentate ventral simple chaeta subdistally serrated, only present on posterior chaetigers (Fig. 1.17). Pharynx extending through 4 chaetigers, with anterior rhomboidal dorsal tooth. Proventriculus extending through 3–4 chaetigers, with 19–22 rows of muscle cells. Pygidium with a pair of cirriform anal cirri.

###### Distribution.

 Africa, Mediterranean, Yellow Sea, Japan, Okhotsk Sea, Bering Sea, North Atlantic, Gulf of Mexico, Belize, Caribbean Sea.

##### Salvatoria limbata

(Claparède, 1868)

http://species-id.net/wiki/Salvatoria_limbata

Figs 1.18–1.21

Salvatoria limbata San Martín 2003:166–169, figs. 82–83.

###### Material examined.

 BMIL197, (2); BMIL498, (4); BMPL197, (3); BMIL197, (7); BMIL297, (5); BMIL498, (2); all specimens associated with *Chondrila nucula*, 1–2 m depth.

###### Description.

 Length to 2.3 mm, width to 0.12 mm. Body small, slender; complete specimens with up to 33 chaetigers. Subulate antennae with enlarged median zone and long distal one. Median antenna longer than lateral ones. Prostomium with a pair of eyespots and two posterior pairs of eyes in trapezoidal arrangement. Compound unidentate falcigers with serrated blades (Fig. 1.18) and with smooth blades 1.19). In each parapodium one bidentate compound falciger with long basal serrations (Fig. 1.20). Slender, unidentate dorsal simple chaeta with slight serrations, from chaetiger 1–2. Ventral simple chaeta unidentate, only present on posterior chaetigers. Acicula subdistally enlarged with pointed tip (Fig. 1.21). Pharynx extending through 3 chaetigers, with dorsal rhomboidal tooth located near anterior margin; proventriculus through 3–4 chaetigers with 16–18 rows of muscle cells. Pygidium with a pair of cirriform anal cirri.

###### Distribution.

*Salvatoria limbata* is considered cosmopolitan, although San Martín (2003) pointed out that it could be restricted to the Northwestern Atlantic Ocean and Mediterranean.

##### Genus Sphaerosyllis Claparède, 1863. Type species: Sphaerosyllis hystrix Claparède, 1863

###### Sphaerosyllis longicauda

Webster & Benedict, 1887

http://species-id.net/wiki/Sphaerosyllis_longicauda

Figs 1.22–1.24

Sphaerosyllis longicauda Webster and Benedict 1887:720, pl. 3, figs. 35–39.
Sphaerosyllis erinaceus Pettibone 1963:135–136, fig. 35a.–Gardiner 1976:131, fig. 10s–v.
Sphaerosyllis longicauda Perkins 1981:1127, figs. 20a–c, 21a–i.–Uebelacker 1984:24–27, fig. 18a–f.–Gobin 2010 (list only).

####### Material examined.

 BMER203, (2), fine sand, 4 m depth; GCPT106, (4), fine sand, 0.5–1.5 m depth.

####### Description.

 Length to 3.1 mm, width to 0.5 mm. Body slender, with up to 26 chaetigers. Papillae scattered, of different length. Prostomium with a pair of eyespots on anterior margin of prostomium, and four large, lentigerous eyes in arc on posterior region of prostomium. Nuchal organs small, rounded, posterior to lateral eyes. Palps wide and short, directed ventrally. Clavate antennae with enlarging basal zone. Tentacular cirri clavate, each with long cirriform dorsal papilla. Dorsal cirri subulate, replaced by a cirriform papilla on chaetiger 2. Dorsal simple chaeta bidentate slender, with slight serrations from chaetiger 1 (Fig. 1.22); dorsal compound falcigers uni and bidentate with serrated blades (Fig. 1.23). Ventral compound falcigers either smooth or with fine serrations. Slender ventral bidentate simple chaeta, only present on posterior chaetigers. Acicula enlarged subdistally, with curved, pointed tip (Fig. 1.24). Pharynx extending through 3 chaetigers, with dorsal tooth located near anterior margin; proventriculus through 3–4 chaetigers with 16–20 rows of muscle cells. Pygidium with a pair of stout anal cirri.

####### Distribution.

 Maine to Florida, Gulf of Mexico, Venezuela.

###### Sphaerosyllis piriferopsis

Perkins, 1981

http://species-id.net/wiki/Sphaerosyllis_piriferopsis

Figs 1.25–1.26

Sphaerosyllis piriferopsis Perkins 1981:1133, figs. 23a–f, 24a–i.–Uebelacker 1984:31–33, fig. 24a–f.–Ruiz-Ramírez and Salazar-Vallejo 2001:130–131, fig. 5(98–107).–San Martín and Bone 2001:613.–Russell 2007:66–69, fig. 7.

####### Material examined.

 PAPC106, (12), coarse sand near *Trypanosyllis testudinum* bed, 0.5–1.3 m depth; BMER103, (8), fine to coarse sand, 1 m depth.

####### Description.

 Length to 2.8 mm, width to 0.15 mm. Body slender, with up to 40 chaetigers. Papillae short scattered. Antennae clavate. Prostomium with two pairs of lentigerous eyes in trapezoidal arrangement. Palps fused dorsally. Dorsal cirri clavate, absent on chaetiger 2, replaced by papillae. Dorsal simple chaeta stout, present on all chaetigers. Dorsal compound falcigers unidentate with serrated blades (Fig. 1.25). Ventral falcigers smooth or with few serrations (Fig. 1.26) Slender ventral simple chaeta, only present on posterior chaetigers. Acicula enlarged subdistally, with curved, pointed tip (Fig. 1.27). Pharynx extending through 3–4 chaetigers, with anterior dorsal tooth. Proventriculus extending through 2 chaetigers, with 10–15 rows of muscle cells. Pygidium with a pair of anal cirri and several dorsal and ventral papillae.

####### Distribution.

 Bahamas, Florida, Gulf of Mexico, Belize, Venezuela.

###### Sphaerosyllis taylori

Perkins, 1981

http://species-id.net/wiki/Sphaerosyllis_taylori

Figs 2.1–2.5

Sphaerosyllis (Sphaerosyllis) taylori Núñez et al. 1992:49–50.
Sphaerosyllis taylori Perkins 1981:1140, fig. 26a–k.–Uebelacker 1984:29–31, fig. 22a–f.–Ruiz-Ramírez and Salazar-Vallejo 2001:131–134, fig. 6(115–122).–San Martín and Bone 2001:614.–San Martín 2003:206–208, fig. 108.–Russell 2007:71–72.

####### Material examined.

 PAPC106, (2), coarse sand, 2 m depth; GCLB104, (8), coarse sand near *Trypanosyllis testudinum* bed, 0.5–1.3 m depth.

####### Description.

 Length to 2.4 mm, width to 0.18 mm. Body broad, with up to 24 chaetigers. Small scattered papillae on dorsum and parapodia. Antennae clavate. Prostomium with two pairs of lentigerous eyes in slightly trapezoidal arrangement. Palps fused. Dorsal cirri clavate, absent from chaetiger 2, replaced by papillae. Dorsal simple chaeta slender, curved (Fig. 2.1), from chaetiger 1, with minute serrations on anterior chaetigers. Anterior dorsal compound falcigers unidentate with serrated edges (Fig. 2.2), posterior ones with few coarse serrations (Fig. 2.3); median and ventral chaetae with smooth blades. Smooth, slender ventral simple chaeta, only present on posterior chaetigers (Fig. 2.4). Acicula stout with curved tip (Fig. 2.5). Pharynx extending through 3 chaetigers, with dorsal tooth located on anterior margin; proventriculus through 2 chaetigers with 17–19 rows of muscle cells. Pygidium with a pair of anal cirri and dorsal papillae.

**Figure 2.**
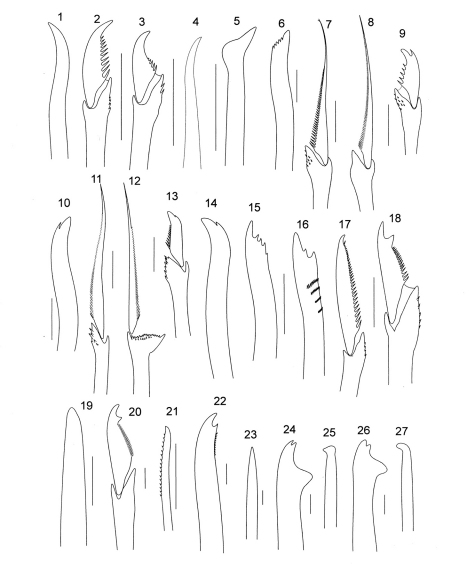
*Sphaerosyllis taylori* **1** dorsal simple chaeta, anterior chaetiger **2** dorsal falciger, anterior chaetiger **3** dorsal falciger, posterior chaetiger **4** ventral simple chaeta, posterior chaetiger **5** acicula from same. *Exogone (Exogone) dispar* **6** dorsal simple chaeta, midbody chaetiger **7** bifid spiniger, anterior chaetiger **8** spiniger, midbody chaetiger **9** bidentate falciger from same **10** ventral simple chaeta, posterior chaetiger. *Exogone (Exogone) lourei*, **11** dorsal spiniger, midbody chaetiger **12** dorsal spiniger, chaetiger 2 **13** falciger, anterior chaetiger **14** ventral simple chaeta, posterior chaetiger. *Parapionosyllis longicirrata* **15** dorsal simple chaeta, anterior chaetiger **16** same, posterior chaetiger **17** dorsal falciger, anterior chaetiger. *Parapionosyllis parvidentata*, dorsal falciger, midbody chaetiger **19** acicula, midbody chaetiger. *Trypanosyllis vittigera* **20** ventral falciger, midbody chaetiger **21** dorsal simple chaeta, posterior chaetiger **22** ventral simple chaeta from same. *Haplosyllis spongicola* **23** acicula, anterior chaetiger **24** simple chaeta, midbody chaetiger **25** acicula from same **26** simple chaeta, posterior chaetiger **27** acicula from same (scale bars: 10µm).

####### Distribution.

 Galapagos Islands, Black Sea, North Atlantic (North Sea to Canary Islands), Mediterranean, Connecticut, Maryland, Florida, Gulf of Mexico, Belize, Venezuela.

##### Genus Exogone Örsted, 1845. Type species: Exogone naidina Örsted, 1845

###### Exogone (Exogone) dispar

(Webster, 1879)

http://species-id.net/wiki/Exogone_(Exogone)_dispar

Figs 2.6–2.10

Paedophylax dispar Webster 1879:223, pl.4, fig. 49, pl.5, figs. 50–55.
Exogone dispar Day1973:33.–Pettibone 1963:130–131, fig. 35d.–Taylor, 1971:201–204.–Westheide 1974:106, figs. 48A–H, 49A–D.–Gardiner 1976:132, fig. llf–i.–Perkins 1981:1090.–Uebelacker 1984:42–43, fig. 36a–e.
Exogone (Exogone) dispar Ruíz-Ramírez and Salazar-Vallejo 2001:127, fig. 3(45–54).–San Martín and Bone 2001:612.–San Martín 2003:274–276, figs. 149, 150. –2005:137–138, figs. 81F, 85A–G.

####### Material examined.

 GCPG198, (4), fine sand, 1 m depth; BMER203, (2), fine sand, 4 m depth; PALB104, (16), sand with *Gemma gemma* (Totten 1834) (Bivalvia: Veneridae), 1 m depth; BMIL197, (6); BMIL498, (12); BMIL602, (3); all specimens associated with *Aplysina fistularis*,1–3 m depth; BMPL197, (12), BMIL602 (8), associated with *Ircinia felix*, 1–2 m depth.

####### Description.

 Length to 6.2 mm, width to 0.4 mm. Body relatively long, with up to 41 chaetigers. Prostomium with two pairs of lentigerous eyes. Median antenna fusiform, lateral antennae small, ovoid. Palps fused dorsally. All cirri ovoid. Dorsal cirri on all chaetigers. Dorsal simple chaeta blunt with subdistal spines (Fig. 2.6), present from chaetiger 1. Dorsal compound pseudospinigers serrated, slightly bifid, on anterior chaetigers (Fig. 2.7), (Fig. 2.8); compound falcigers bidentate with small distal tooth, and spines on the shaft-head (Fig. 2.9). Ventral simple chaeta bidentate with small distal tooth, only present on posterior chaetigers (Fig. 2.10). Pharynx extending through 4 chaetigers, with marginal crown of papillae, and subterminal dorsal tooth. Proventriculus extending through 4 chaetigers, with 19–22 rows of muscle cells. Pygidium with a pair of cirriform, relatively long cirri.

####### Remarks.

 From hard bottom substrats of Trinidad and Tobago islands is the most abundant species (Gobin 2010).

####### Distribution.

 North Pacific, Galapagos Islands, South Japan, Australia, North Atlantic, Mediterranean, Arctic, Alaska to Mexico, South Africa, Maine to Florida, Gulf of Mexico, Trinidad & Tobago, Venezuela.

###### Exogone (Exogone) lourei

Berkeley & Berkeley, 1938

http://species-id.net/wiki/Exogone_(Exogone)_lourei

Figs 2.11–2.14

Exogone lourei Berkeley and Berkeley 1938 :44, figs. 6–12.–Banse 1972:200, fig. 5A–D.–Banse and Hobson 1974: 58, fig. 14h–j.–Perkins 1981:1092.–Uebelacker 1984:39–41, fig.34a–f.–Russell 2007:56–57, fig. 2.–Gobin 2010 (list only).
Exogone (Exogone) lourei San Martín and Bone 2001:612. –San Martín 2005:129–130, fig. 78A–J.

####### Material examined.

 GCPG198, (3), fine sand, 2 m depth; BMER203; (15); fine sand, 4 m depth; BMC101, (7); BMLV101, (9), inside dead *Millepora alcicornis*, 1–2 m depth; BMC103 (7), medium sand 1–2 m depth.

####### Description.

 Length to 7.6 mm, width to 0.2 mm. Body with up to 50 chaetigers. Prostomium with two pairs of lentigerous eyes. Median antenna digitiform, lateral antennae short, ovoid. Palps fused dorsally. Tentacular, dorsal and ventral cirri ovoid. Dorsal simple chaeta with bent tip, minutely serrated on outer edge, present from chaetiger 1. Dorsal compound spinigers serrated (Fig. 2.11), with shaft-heads enlarged on chaetiger 2 (Fig. 2.12); ventral compound falcigers on anterior chaetigers bidentate with very small terminal tooth and serrated edges (Fig. 2.13). Ventral simple chaeta bidentate (Fig. 2.14), present on middle and posterior chaetigers. Pharynx extending through 4–5 chaetigers, with 10 marginal papillae and subterminal dorsal tooth. Proventriculus extending from chaetigers 4–5 to 5–8, with 17–24 rows of muscle cells. Pygidium with a pair of anal cirri.

####### Distribution.

 South of British Columbia to Panama, Canary Islands, Australia, Florida, Gulf of Mexico, Belize, Cuba, Venezuela.

##### Genus Parapionosyllis Fauvel, 1923. Type species: Pionosyllis gestans Pierantoni, 1903

###### Parapionosyllis longicirrata

(Webster & Benedict, 1884)

http://species-id.net/wiki/Parapionosyllis_longicirrata

Figs 2.15–2.17

Sphaerosyllis longicirrata Webster and Benedict 1884:715, pl. 8, figs. 95–100.
Parapionosyllis longicirrata Pettibone 1963:132, fig. 35e,f.–Perkins 1981:1102, fig. 9a–m.–Uebelacker 1984:58–60, fig. 52a–g.

####### Material examined.

 PAPC106, (13), as epibionts on tubes of *Americonuphis magna* (Andrews 1891), 0.3–0.6 m depth.

####### Description.

 Length to 3.9 mm, width to 0.26 mm. Body with up to 42 chaetigers. Prostomium with a pair of anterior eyespots and two pairs of posterior lentigerous eyes in trapezoidal arrangement. Antennae fusiform with digitiform end. Palps fused dorsally over half their length. Tentacular and dorsal cirri subulate. Dorsal cirri fusiform. Dorsal simple chaeta with subdistal serrations present from chaetiger 1 (Fig. 2.15, 2.16). Compound falcigers unidentate with coarse serrations and subdistal spine (Fig. 2.17). Ventral simple falcate chaeta, only present on posterior chaetigers. Acicula with circular end. Pharynx extending through 3 chaetigers, with anterior middorsal tooth. Proventriculus extending through 2 chaetigers, with 14–17 rows of muscle cells. Pygidium with two cirriform anal cirri.

####### Distribution.

 Massachusetts, Florida, Gulf of Mexico, Venezuela.

#### Subfamily Syllinae Grube, 1850. Genus Trypanosyllis Claparède, 1864. Type species: Syllis zebra Grube, 1860

##### Trypanosyllis parvidentata

Perkins, 1981

http://species-id.net/wiki/Trypanosyllis_parvidentata

Figs 2.18–2.19

Trypanosyllis parvidentata Perkins 1981:1161, fig. 36a–h.–Uebelacker 1984:91–93, fig. 86a–e.

###### Material examined.

 Guacarapo (Gulf of Cariaco), GCPG198, (1), fine sand, 1 m depth.

###### Description.

 Length to 9.4 mm, width to 0.65 mm. Body incomplete, with up to 88 chaetigers. Prostomium with a pair of lentigerous eyes in trapezoidal arrangement. Median antenna with 10 articles, lateral antennae with 9 articles. Palps separated. Dorsal tentacular cirri with 11 articles, ventral ones with 9 articles. Dorsal cirri with 13 articles in chaetiger 1; from chaetiger 5 alternating longer, with 15 articles, and shorter cirri, with 9 articles. Dorsal compound falcigers bidentate, serrated (Fig. 2.18), ventral ones with shorter blades. Ventral simple chaeta bidentate, slender, on posterior chaetigers. Acicula stout, solitary in posterior chaetigers (Fig. 2.19) Pharynx extending through 5 chaetigers. Proventriculus extending through 4 chaetigers, with 17 rows of muscle cells.

###### Distribution.

 Southern Florida, Gulf of Mexico, West Indies, Venezuela.

##### Trypanosyllis vittigera

Ehlers, 1887

http://species-id.net/wiki/Trypanosyllis_vittigera

Figs 2.20–2.22

Trypanosyllis vittigera Hartman 1951:41.–Uebelacker 1984:88, fig. 82a–h.–San Martín 1991:227–228.

###### Material examined.

 GCPG198, (4), fine sand, 1 m depth; GCPT106, (8), fine sand, 0.5–1.5 m depth.

###### Description.

 Length to 12.4 mm, width to 2.2 mm, with 56 chaetigers. Body long, and flattened; with two dorsal transverse brown stripes per segment anteriorly. Prostomium with a pair of lentigerous eyes in trapezoidal arrangement. Median antenna with 17–21 articles, lateral ones with 10–13 articles. Dorsal tentacular cirri with 29–36 articles, lateral ones with 19–23 articles. Dorsal cirri alternating in length longer, with 19–26 articles, and shorter, with 9–12 articles. Ventral cirri cirriform. Compound falcigers bidentate with small serrations, blades of dorsal falcigers longer than ventral ones (Fig. 2.20). Dorsal simple chaeta slender with subdistal small serrations (Fig. 2.21), and ventral simple chaeta bidentate, with subdistal serrations (Fig. 2.22), both only present on posterior chaetigers. Pharynx extending through 4–5 chaetigers, with distal trepan of ten teeth. Proventriculus extending through 4 chaetigers, with 31–37 rows of muscle cells. Pygidium with a pair of anal cirri with 6–9 articles.

###### Remarks.

 According to San Martín (1991) *Trypanosyllis zebra* from the Mediterranean Sea is very similar to *Trypanosyllis vittigera*, differing only in the number of teeth on the trepan and in the length of the proventriculus, thus suggesting the need for a revision of *Trypanosyllis zebra* and related species.

###### Distribution.

 Circumtropical.

##### Genus Haplosyllis Langerhans, 1879. Type species: Syllis spongicola Grube, 1855

###### Haplosyllis spongicola

(Grube, 1855)

http://species-id.net/wiki/Haplosyllis_spongicola

Figs 2.23–2.28

Syllis (Haplosyllis) spongicola Fauvel 1923:257, fig. 95a–d.
Haplosyllis spongicola Imajima 1966:220, fig. 38a–h.
Syllis (Haplosyllis) spongicola Day 1967:240–241, fig. l2.1.e–i.–Gardiner 1976:139, fig. 12i–k.
Haplosyllis spongicola Uebelacker 1984:109–111, fig. 104a–d.–San Martín 1991:233.–San Martín and Bone 2001:615.–San Martín 2003:323–325, figs. 179–180.–Martin et al. 2003:145–162, figs. 1–12.–Lattig et al. 2007:554–557, figs. 1–2.–Gobin 2010 (list only).

####### Material examined:

 BMIL598, (84); BMPL498, (36); associated with *Chondrila nucula*, 1–2 m depth; BMIL297, (1566); BMIL498, (607); BMPL197, (132); BMPL297, (506), all specimens associated with *Aplysina fistularis*, 1–3 m depth; BMIL297, (1147); (3789), BMIL397; BMIL498, (3689). BMPL297, (2852); BMPL297, (2667), BMPL398, (2808); all specimens associated with *Ircinia felix*, 1–2 m depth; GCPG198, (78), fine sand, 1 m depth; GCET103, (18); BMER103, (13), fine to coarse sand, 1 m depth. BMMQ205, (2), inside dead *Millepora alcicornis*, 1–2 m depth.

####### Description.

 Length to 5.6 mm,width 2.4 mm. Body with up to 85 chaetigers, broad anteriorly, thinner from mid-body to pygidium. Prostomium with a pair of small eyes in trapezoidal arrangement. Median antenna with 24–33 articles, lateral antennae with 8–19 articles. Palps fused dorsally. Dorsal tentacular cirri with 18–36 articles. Anterior dorsal cirri with 7–47 articles. First cirri longer than remaining ones. Dorsal cirri of middle region alternating in length longer, with 8–16 articles, and shorter, with 4–10 articles. Ventral cirri digitiform, shorter than parapodial lobes. Two (1–3) simple and stout chaetae, with two distal teeth and main fang prominent (Figs. 2.24, 2.26) and with upper side rugose. Aciculae either with pointed tip (Fig. 2.23) or with curved end (Figs. 2.25, 2.27). Pharynx extending through 7–11 chaetigers; with 8–10 soft distal papillae, encircling middorsal tooth. Proventriculus extending through about 12 chaetigers, with 34–52 rows of muscle cells. Pygidium with a pair of long moniliform anal cirri.

####### Remarks.

 This species is one of the most abundant syllid in the Great Caribbean region in both soft and hard bottoms (Granados-Barba et al. 2003); Gobin (2010) pointed out that is one of the most abundant species from hard bottoms of Trinidad and Tobago. Martin et al. (2003) based on the wide variability observed within the *Haplosyllis* species, pointed out that the so-called *Haplosyllis spongicola* must be considered as a pseudo-sibling species-complex.

####### Distribution.

 Considered cosmopolitan, although Martin et al.(2003) and Lattig et al. (2007) pointed out that records in temperate and tropical seas must be reviewed.

##### Genus Opisthosyllis Langerhans, 1879. Type species: Opisthosyllis brunnea Langerhans, 1879

###### Opisthosyllis sp.

Figs 3.1–3.7

####### Material examined.

 IMLR102, (1); IMLR202, (4), as epibionts on *Crassostrea rhizophorae*, 0–0.5 m.

####### Description.

 Length up to 21 mm, 0.2 mm in width, with 78 chaetigers. Prostomium with two pairs of lentigerous eyes in trapezoidal arrangement on posterior half of prostomium. Antennae longer than prostomium and palps, with 18–19 articles each. Palps separated at the base. Dorsal tentacular cirri with 26 articles, ventral ones with 18. Dorsal cirri, alternating in length longer, with 23–25 articles, and shorter, with 17–19 articles (Fig. 3.1). Compound falcigers unidentate with spiniferous cutting edge, dorsal ones with longer blades (Fig. 3.2) than ventral ones (Fig. 3.3, 3.4). Acicula subterminally thickened (Fig. 3.5, 3.6). Dorsal simple chaeta with slightly bifid tip (Fig. 3.7), only present on last chaetigers. Pharynx extending through 9 chaetigers; with dorsal tooth located at the level of the chaetiger 7; proventriculus extending through 6–7 chaetigers, with about 45 rows of muscle cells.

**Figure 3.**
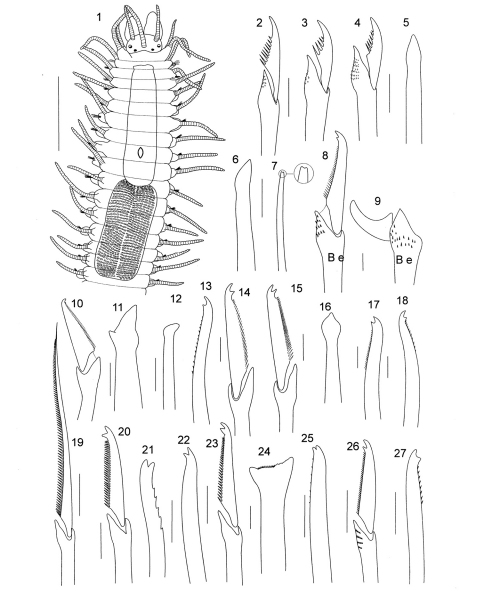
*Opisthosyllis* sp. **1** anterior end, dorsal view **2** dorsal long falciger **3, 4** ventral short falcigers **5, 6** aciculae **7** dorsal simple chaeta, posterior chaetiger. *Branchiosyllis exilis* **8** dorsal falciger, anterior chaetiger **9** ventral falciger, midbody chaetiger. *Syllis amica* **10** falciger, anterior chaetiger **11** dorsal simple chaeta, midbody chaetiger **12** acicula from same. *Syllis armillaris* **13** dorsal simple chaeta, posterior chaetiger **14** bidentate falciger, anterior chaetiger. *Syllis coralicolla* **15** bidentate falciger, anterior chaetiger **16** acicula, posterior chaetiger **17** dorsal simple chaeta from same **18** ventral simple chaeta from same. *Syllis cornuta* **19** dorsal spiniger, anterior chaetiger **20** dorsal bidentate falciger from same **21** dorsal simple chaeta, posterior chaetiger **22** ventral simple chaeta from same. *Syllis gracilis* **23** dorsal falciger, anterior chaetiger **24** ypsiloid simple chaeta, midbody chaetiger. *Syllis prolifera* **25** dorsal simple chaeta, posterior chaetiger **26** dorsal falciger, anterior chaetiger **27** ventral simple chaeta, posterior chaetiger (scale bars: 1 = 1 mm; 2–27 = 10µm).

####### Remarks.

*Opisthosyllis* sp. resembles *Opisthosyllis brunnea* Langerhans 1991, in having compound chaetae with unidentate blades, and in the location of the pharyngeal tooth; it differs in the absence of an occipital flap, having a wider proventriculus, and in the number of rows of muscle cells in the proventriculus.

##### Genus Branchiosyllis Ehlers, 1887. Type species: Branchiosyllis oculata Ehlers, 1887

###### Branchiosyllis exilis

(Gravier, 1900)

http://species-id.net/wiki/Branchiosyllis_exilis

Figs 3.8–3.9

Syllis (Typosyllis) exilis Gravier 1900:160, pl. 10, fig. 19.
Branchiosyllis exilis , Westheide 1974:60, fig. 26A–H.–Uebelacker 1984:105–107, fig. 100a–f.–San Martín 1991:233.–San Martín and Bone 2001:614.–San Martín 2003:332–336, figs. 184–185.

####### Material examined.

 BMIL197, (1); (9), BMIL297, (9); BMIL498, (8); BMPL197, (7); BMPL398, (16), all specimens associated with *Chondrila nucula*; BMIL498, (11); (22); BMPL197, (5); BMPL398, (6), all specimens associated with *Aplysina fistularis*, 1–3 m depth; BMPL197, (13); BMIL498, (14); BMPL197, (6), all specimens associated with *Ircinia felix*, 1–2 m depth.

####### Description.

 Length to 17.6 mm,width 1.2 mm. Body with up to 68 chaetigers. Prostomium with a pair of anterior small eyes and two posterior pairs of lentigerous eyes in trapezoidal arrangement. Median antenna with 15–21 articles; lateral antennae with 12–15 articles. Palps stout, rounded, fused basally. Dorsal tentacular cirri with 19–23 articles, ventral ones with 10–13 articles. Anterior dorsal cirri alternating longer, with 22–38 articles, and shorter, with 17–31 articles. Length and number of articles diminishing posteriorly. Dorsal compound falcigers bidentate (Fig. 3.8), ventral falcigers bidentate in anterior chaetigers, falcate in middle and posterior chaetigers (Fig. 3.9). Pharynx extending through 7 chaetigers. Proventriculus extending through 9–12 chaetigers, with 39–48 rows of muscle cells. Pygidium with a pair of anal cirri with 13–21 articles.

####### Distribution.

 Circumtropical, Mediterranean, Baleares and Chafarinas Island (Spain), and Venezuela.

##### Genus Syllis Lamarck, 1818. Type species: Syllis monilaris Savigny in Lamarck, 1818

###### Syllis amica

Quatrefages, 1865

http://species-id.net/wiki/Syllis_amica

Figs 3.10–3.12

Syllis (Typosyllis) amica Uebelacker 1984:127–129, fig. 120a–e.
Syllis amica Fauvel 1923:258–259, fig. 95e–n.–San Martín 2003:366–370, figs. 199–200.

####### Material examined.

 GCPG198, (15), fine sand, 1 m depth; PAPC106, (2), coarse sand, 2 m depth; GCLB205, (6), fine sand, 1 m depth; (8), GCLB205, coarse sand near *Thalassia testudinum* bed, 0.5–1.3 m depth; BMC101, (4), medium sand, 1 m depth; BMLV101, (28), inside dead *Millepora alcicornis*, 1–2 m depth; BMIL297, (11); BMIL498, (6), associated with *Aplysina fistularis*, 1–3 m depth; BMPL297, (15); BMPL398, (8), associated with *Ircinia felix*, 1–2 m depth; BMIL397, (34), associated with *Chondrila nucula*, 1–2 m depth.

####### Description.

 Length to 22.6 mm,width 0.2 mm. Body with up to 125 chaetigers. Prostomium with two pairs of lentigerous eyes. Median antenna with 15–20 articles, lateral antennae with 12–19 articles. Dorsal tentacular cirri with 16–21 articles, ventral ones with 9–12 articles. Dorsal cirri of chaetiger 1 longer than remaining, with 19–22 articles. Dorsal cirri alternating longer, with 19–22 articles, and shorter, with 15–18 articles in middle region. Compound falcigers unidentate, coarsely serrated (Fig. 3.10). Dorsal chaeta in middle and posterior regions, simple due to loss of blade (Fig. 3.11). Dorsal and ventral simple chaetae of posterior region, slender, bidentate and minutely serrated. Acicula distally enlarged (Fig. 3.12). Pharynx extending through 6–8 chaetigers, with middorsal anterior tooth. Proventriculus extending through 4–5 chaetigers, with about 40 rows of muscle cells. Pygidium with a pair of anal cirri moniliform and digitiform, short cirrus.

####### Distribution.

 Cosmopolitan in temperate and tropical seas.

###### Syllis armillaris

(Müller, 1771)

http://species-id.net/wiki/Syllis_armillaris

Figs 3.13–3.14

Nereis armillaris Müller 1771 in Müller 1776:217.*Syllis (Typosyllis) armillaris*Fauvel 1923:264, fig. 99a–f.**–**Day 1967:249, fig. 12.4.a–d.–Uebelacker 1984:129–131, 122a–g.
Syllis armillaris San Martín 2003:423–426, figs. 232–233.

####### Material examined.

 BMIL197, (2); BMIL602, (9), associated with *Aplysina fistularis*, 1–3 m depth; BMIL197, (4); BMIL397 (6), associated with *Ircinia felix*, 1–2 m depth.

####### Description.

 Length to 11.5 mm,width 0.62 mm. Body with up to 121 chaetigers. Prostomium with a pair of anterior eyespots and two pairs of lentigerous eyes in trapezoidal arrangement. Median antenna with 9–19 articles, lateral antennae with 11–13 articles. Palps fused basally. Dorsal tentacular cirri with 11–16 articles, ventral ones with 9–15 articles. Dorsal cirri alternating in anterior segments longer, with 12–16 articles, and shorter, with 8–10 articles, becoming shorter in middle and posterior regions. Slender, bidentate dorsal simple chaeta (Fig. 3.13) only present on posterior chaetigers. Compound falcigers bidentate in anterior (Fig. 3.14) and posterior chaetigers, unidentate or subbidentate in midbody region. Simple ventral chaeta bidentate only present on posterior chaetigers. Pharynx extending through 8–9 chaetigers, with 10 marginal papillae encircling middorsal tooth. Proventriculus extending through 6 chaetigers, with 36–44 rows of muscle cells. Pygidium with a pair of anal cirri with 14–18 articles and slender midventral cirrus.

####### Distribution.

 Cosmopolitan.

###### Syllis coralicolla

Verrill, 1900

http://species-id.net/wiki/Syllis_coralicolla

Figs 3.15–3.18

Syllis coralicolla San Martín 1992:185–186, fig. 1A–D.–San Martín and Bone 2001:616.–Nogueira and San Martín 2002:73–75, figs. 11, 12.–San Martín 2003:439–443, figs. 242–243.

####### Material examined.

 BMC101, (5), inside dead *Millepora alcicornis*, 1–2 m depth.

####### Description.

 Length to 9.5 mm,width 0.59 mm. Body with up to 79 chaetigers. Prostomium with a pair of anterior eyespots and two pairs of lentigerous eyes in trapezoidal arrangement. Median antenna with 19–30 articles, lateral ones with 15–17 articles. Dorsal tentacular cirri with 24–38 articles, ventral ones with 15–18 articles. Dorsal cirri of chaetiger 1 long, with 35–41 articles. Dorsal cirri alternating longer, with 24–33 articles, and shorter, with 14–19 articles. Compound falcigers bidentate and serrated (Fig. 3.15). Acicula enlarged subdistally with rounded tip (Fig. 3.16). Dorsal bifid simple chaeta (Fig. 3.17), and ventral simple chaeta bidentate, both only present on posterior chaetigers (Fig. 3.18). Pharynx extending through 7–8 chaetigers, with anterior tooth. Proventriculus extending through 5–7 chaetigers, with 33–41 rows of muscle cells. Pygidium with a pair of anal cirri.

####### Distribution.

 Iberian Peninsula, Mediterranean, Balear Islands, Antillas, Bermuda, Cuba, Venezuela.

###### Syllis cornuta

Rathke, 1843

http://species-id.net/wiki/Syllis_cornuta

Figs 3.19–3.22

Syllis (Ehlersia) cornuta Fauvel 1923:267, fig. 100g–1.
Langerhansia cornuta Imajima 1966:256, fig. 51a–o.
Syllis (Langerhansia) cornuta Day 1967:244, fig. 12.2.s–u; 1973:29.–Gardiner 1976:140, fig. 12o–s.–Uebelacker 1984:120–122, fig. 114a–f.
Syllis cornuta Pettibone 1963:118, figs. 31i, j.

####### Material examined.

 BMIL297, (37); (2), BMIL397; BMIL498, (18), all specimens associated with *Aplysina fistularis*, 1–3 m depth; BMPL297, (10); BMPL398, (3), all specimens associated with *Ircinia felix* 1–2 m depth.

####### Description.

 Length to 29.5 mm,width 0.77 mm. Body with up to 107 chaetigers. Prostomium with a pair of anterior eyespots and two pairs of eyes in trapezoidal arrangement. Median antenna with 11–27 articles; lateral ones with 9–20 articles. Palps long, fused basally. Dorsal tentacular cirri with 11–19 articles, ventral ones with 7–16 articles. Dorsal cirri on anterior chaetigers with 7–33 articles, 5–25 articles medially. Dorsal compound spiniger chaetae, finely serrated, present from chaetiger 1 (Fig. 3.19). Compound falcigers bidentate, serrated with small subterminal tooth (Fig. 3.20). Stout, bifid, dorsal simple chaeta with subdistal serrations (Fig. 3.21) and bidentate ventral simple chaeta (Fig. 3.22) both only present on posterior chaetigers. Pharynx extending through 6–12 chaetigers, with a crown of ten soft papillae and middorsal subterminal tooth. Proventriculus extending through 4–6 chaetigers, with 36–48 rows of muscle cells. Pygidium with a pair of anal cirri with 18–28 articles and midventral digitiform cirrus.

####### Distribution.

 Cosmopolitan.

###### Syllis gracilis

Fauvel, 1923

http://species-id.net/wiki/Syllis_gracilis

Figs 3.23–3.24

Syllis (Syllis) gracilis Day 1967:241, fig. 12.1.m–p.–Gardiner 1976:139, fig. 12 l–n.–Uebelacker 1984:116–118, fig. 112a–h.–Nogueira and San Martín 2002:68–72, figs. 7, 8.
Syllis gracilis Fauvel 1923:259 fig. 96f–i.–Pettibone 1963:116, fig. 32a–e.–Imajima 1966:248, fig. 49a–k.– Taylor, 1971:212–214.–San Martín 1992:178.–2003:413–416, figs. 226–227.–Gobin 2010 (list only).

####### Material examined.

 BMPL297, (8); BMPL398, (4), associated with *Chondrila nucula*, 1–2 m depth; BMPL297, (2); BMPL398, (4), all specimens associated with *Aplysina fistularis*, 1–3 m depth; BMIL598, (4); BMPL297, (3); BMPL398, (16); all specimens associated with *Ircinia felix*, 1–2 m depth; GCET1030, (6), fine to coarse sand, 1 m depth.

####### Description.

 Length to 19.5 mm,width 0.7 mm. Body with up to 107 chaetigers. Anterior segments with a pair of dark, dorsal, transverse stripes. Prostomium with two pairs of lentigerous eyes in trapezoidal arrangement. Median antenna with 14–20 articles; lateral antennae with 12–15 articles. Dorsal tentacular cirri with 12–18 articles, ventral ones with 6–12 articles. Dorsal cirri alternating longer, with 16–21 articles, and shorter, with 14–16 articles, diminishing posteriorly. Anterior chaetae compound, bidentate, falcigers (Fig. 3.23), replaced by thick, simple, ypsiloid chaetae (Fig. 3.24) on median chaetigers. Posterior chaetae compound, bidentate, falcigers and slender, bidentate dorsal and ventral simple chaetae. Acicula enlarged subdistally. Pharynx extending through 8–9 chaetigers, with 10 marginal soft papillae, and middorsal subdistal tooth. Proventriculus extending through 11–13 chaetigers, with 36–42 rows of muscle cells. Pygidium with a pair of anal cirri with 6–12 articles and midventral cirrus.

####### Distribution.

 Cosmopolitan in temperate and tropical seas.

###### Syllis prolifera

Krohn, 1852

http://species-id.net/wiki/Syllis_prolifera

Figs 3.25–3.27

Typosyllis prolifera Imajima 1966:292, fig. 65a–n.
Syllis (Typosyllis) prolifera Fauvel 1923:261, fig. 97a–g.–Day 1967:248, fig. 12.3.g–i.– 1973:30.–Uebelacker 1984:150–151, fig. 146a–g.
Syllis prolifera San Martín 1992:171–173, fig. 1E–H.–San Martín and Bone 2001:617.–San Martín 2003:344–347, figs. 186–187.–Gobin 2010 (list only).

####### Material examined:

 BMIL297, (3); BMPL297, (3); BMPL398, (10), BMIL598, (8); all specimens associated with *Chondrila nucula*, 1–2 m depth.

####### Description.

 Length to 25.5 mm,width 0.7 mm. Body with up to 97 chaetigers. Anterior segments with a pair of brown, dorsal, transverse stripes. Prostomium with a pair of anterior eyespots and two pairs of eyes in trapezoidal arrangement. Median antenna with 21–30 articles, lateral ones with 17–23 articles. Dorsal tentacular cirri with 14–28 articles, ventral ones with 8–14 articles. Dorsal cirri alternating longer, with 24–42 articles, and shorter, with 10–19 articles. Dorsal simple chaeta, bifid from mid-body chaetigers (Fig. 3.25). Compound falcigers bidentate and serrated (Fig. 3.26). Ventral simple chaeta bidentate, slender with subdistal serrations (Fig. 3.27), only present on posterior chaetigers. Anterior aciculae slender with blunt tip, posterior ones enlarged distally. Pharynx extending through 5 chaetigers, with 10 marginal papillae encircling middorsal tooth. Proventriculus extending through 5 chaetigers, with 27–35 rows of muscle cells. Pygidium with a pair of anal cirri with 14–29 articles and digitiform midventral cirrus.

####### Distribution.

 Japan, Indian Ocean, South Africa, Mediterranean Sea, Atlantic Ocean, Caribbean Sea, Gulf of Mexico.

###### Syllis pseudoarmillaris

Nogueira & San Martín, 2002

http://species-id.net/wiki/Syllis_pseudoarmillaris

Figs 4.1–4.5

Syllis pseudoarmillaris Nogueira and San Martín 2002:83–85, figs. 17–18.

####### Material examined.

 PAPC106, (8), as epibionts on *Americonuphis magna* tubes (Andrews 1891), 0.3–0.6 m depth; GCLB205 (3), fine sand, 1 m depth.

####### Description:

 Length to 9.5 mm,width 0.29 mm. Body with up to 91 chaetigers. Prostomium with two pairs of eyes in trapezoidal arrangement. Antennae moniliform; median antenna with 13–19 articles; lateral ones with 8–12 articles. Palps basally fused. Dorsal tentacular cirri with 14–19 articles, ventral ones with 9–11 articles. Dorsal cirri moniliform, those from chaetiger 1 longer than the following ones, with 15–21 articles; dorsal midbody chaetigers with 9–14 articles, posterior ones with few articles (3–6). Compound falcigers bidentate, with serrated blades (Fig. 4.1). Dorsal and ventral simple chaetae bidentate with subdistal serrations (Fig. 4.2, 4.3) on posterior chaetigers. Anterior acicula subdistally enlarged with blunt tip (Fig. 4.4), posterior acicula subdistally enlarged with bent tip (fig. 4.5). Pharynx extending through 7–9 chaetigers, with distal middorsal large tooth; surrounded by soft papillae; proventriculus extending through 4 chaetigers**,** with 38–41 rows of muscle cells. Pygidium with a pair of anal cirri with 3–5 articles.

**Figure 4.**
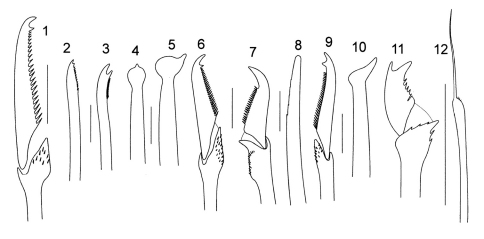
*Syllis pseudoarmillaris* **1** bidentate falciger, anterior chaetiger **2** dorsal simple chaeta, posterior chaetiger **3** ventral simple chaeta from same **4** acicula, anterior chaetiger **5** acicula, posterior chaetiger. *Syllis vittata* **6** dorsal falciger, anterior chaetiger **7** falciger, posterior chaetiger. *Parasphaerosyllis indica* **8** dorsal simple chaeta, midbody chaetiger **9** dorsal falciger, anterior chaetiger **10** acicula, midbody chaetiger. *Myrianida convoluta* **11** bidentate falciger, anterior chaetiger **12** bayonet chaeta, midbody chaetiger (scale bars: 10µm).

####### Distribution.

 Brazil, Venezuela.

###### Syllis vittata

Grube, 1840

http://species-id.net/wiki/Syllis_vittata

Figs 4.6–4.7

Syllis (Typosyllis) vittata Fauvel 1923:263–264, fig. 98i–l.–Day 1967:252, Fig. 12.4.m–o.
Syllis vittata Taylor, 1971:220–222.–San Martín 2003:430–432, figs. 236–237.

####### Material examined.

 BMIL803, (8), on rocks covered by *Enteromorpha intestinalis* (Linnaeus), 1–2 m depth.

####### Description.

 Length to 21.5 mm,width 1.1 mm. Body broad with up to 101 chaetigers, with dorsal transverse dark stripe per segment. Prostomium with two pairs of eyes in trapezoidal arrangement. Median antenna with 20–26 articles; lateral ones with 18–24 articles. Palps stout basally fused. Antennae moniliform; median antenna with 23–31 articles, lateral antennae with 20–23 articles. Dorsal tentacular cirri with 30–39 articles, ventral ones with 19–23 articles. Dorsal cirri alternating longer, with 20–27 articles, and shorter, with 19–24 articles. Dorsal simple chaeta bifid serrated, only present on posterior chaetigers. Anterior compound falcigers bidentate and serrated (Fig. 4.6). Posterior compound falcigers with short blades unidentate and serrated (Fig. 4.7). Ventral simple bidentate serrated chaeta, only present on posterior chaetigers. Pharynx extending through 10 chaetigers, with 10 marginal papillae encircling distal tooth. Proventriculus extending through 9 chaetigers, with 37–40 rows of muscle cells. Pygidium with a pair of anal cirri with 9–12 articles and digitiform midventral cirrus.

####### Distribution.

 Eastern Atlantic, Mediterranean Sea, Indian Ocean, Florida, Great Caribbean.

##### Genus Parasphaeropsyllis Monro, 1937. Type species: Parasphaeropsyllis indica Monro, 1937

###### Parasphaeropsyllis indica

Monro, 1937

http://species-id.net/wiki/Parasphaeropsyllis_indica

Figs 4.8–4.10

Parasphaeropsyllis indica Rioja 1958:246–251, figs. 21, 22, 26, 27.–Westheide 1974:64–66, figs. 27–29.–San Martín 1991:234.–1994:130.

####### Material examined.

 BMMQ205, (19); BMLV101, (8); GCET103, (22), inside dead *Millepora alcicornis*, 1–2 m depth; BMPL398, (24), associated with *Aplysina fistularis*, 1–3 m depth.

####### Description.

 Length to 9.5 mm,width 0.6 mm. Body slender with up to 106 chaetigers. Prostomium with two pairs of lentigerous eyes in trapezoidal arrangement. Median antenna with 18–21 articles, lateral ones with 13–15 articles. Dorsal tentacular cirri with 32–51 articles, ventral ones with 18–23 articles. Dorsal cirri alternating longer, with 21–33 articles, and shorter, with 19–22 articles. Large, ovoid dorsal cirri with small distal button, from chaetigers 24. Dorsal simple chaeta with blunt end and subdistally serrated (Fig. 4.8). Compound falcigers bidentate and serrated (Fig. 4.9). Ventral simple chaeta bidentate, slender. Acicula subdistally enlarged, with acuminate, oblique tip (Fig. 4.10). Pharynx extending through 6 chaetigers, with 10 marginal papillae encircling anterior tooth. Proventriculus extending through 3–5 chaetigers, with 25–28 rows of muscle cells.

####### Distribution.

 Circumtropical.

#### Subfamily Autolytinae Langerhans, 1879. Genus Myrianida Milne Edwards, 1845. Type species: Myrianida fasciata Milne Edwards, 1845

##### Myrianida convoluta

(Cognetti, 1953)

http://species-id.net/wiki/Myrianida_convoluta

Figs 4.11–4.12

Autolytus convolutus Cognetti 1953:323–332, figs 1–12.–1957:71–72, fig. 15A–B.– Ben-Eliahu 1972: 217–218, fig. 14A–D.–Amaral and Nonato 1975: 235–236.– Ben-Eliahu 1977: 85–86, fig. 12.–San Martín 1994:271.
Autolytus (Regulatus) convolutus Imajima 1966:47–49, fig. 12A–H.
Autolytus convolutus San Martín 2003:483–486, figs 265–266.
Myrianida convoluta Nygren 2004:125–126, fig. 60A–D.

###### Material examined.

 GCPG198, (2), on artificial substrate (PVC pipe), 1 m depth; BMC101, (1), inside dead *Millepora alcicornis*, 1–2 m depth.

###### Description.

 Length to 2.6 mm,width 0.2 mm. Body slender with up to 14 chaetigers, without stolons. Prostomium with a pair of anterior eyespots and two posterior pairs of lentigerous eyes in trapezoidal arrangement. Median antenna longer than lateral ones. Palps fused. Nuchal organs extending to chaetiger 2. Dorsal tentacular cirri as long as lateral antennae; ventral ones shorter. Dorsal cirri of chaetiger 1 as long as median antenna, remaining dorsal cirri short, digitiform. Compound bidentate chaetae with short serrated blades, with small distal tooth and broad subdistal one (Fig. 4.11). Slender bayonet chaetae from chaetiger 4–11 (Fig. 4.12). Pharynx with many circumvolutions. Trepan with 9 equal teeth. Proventriculus in chaetigers 8–10 with 19–20 rows of muscle cells. Pygidium with a pair of anal cirri.

###### Distribution.

 North Pacific, Suez Canal, Japan, North Atlantic, Mediterranean, Great Caribbean.

## Supplementary Material

XML Treatment for Odontosyllis enopla

XML Treatment for Odontosyllis guillermoi

XML Treatment for Syllides floridanus

XML Treatment for Salvatoria clavata

XML Treatment for Salvatoria limbata

XML Treatment for Sphaerosyllis longicauda

XML Treatment for Sphaerosyllis piriferopsis

XML Treatment for Sphaerosyllis taylori

XML Treatment for Exogone (Exogone) dispar

XML Treatment for Exogone (Exogone) lourei

XML Treatment for Parapionosyllis longicirrata

XML Treatment for Trypanosyllis parvidentata

XML Treatment for Trypanosyllis vittigera

XML Treatment for Haplosyllis spongicola

XML Treatment for Opisthosyllis

XML Treatment for Branchiosyllis exilis

XML Treatment for Syllis amica

XML Treatment for Syllis armillaris

XML Treatment for Syllis coralicolla

XML Treatment for Syllis cornuta

XML Treatment for Syllis gracilis

XML Treatment for Syllis prolifera

XML Treatment for Syllis pseudoarmillaris

XML Treatment for Syllis vittata

XML Treatment for Parasphaeropsyllis indica

XML Treatment for Myrianida convoluta
